# How life circumstances during public health crises affect people to share and correct misinformation: a perspective of the third-person effect

**DOI:** 10.3389/fpsyg.2024.1418504

**Published:** 2024-10-16

**Authors:** Xiang Tian

**Affiliations:** School of Media and Communication, Shanghai Jiao Tong University, Shanghai, China

**Keywords:** misinformation, third-person effect, COVID-19, health communication, Shanghai’s lockdown

## Abstract

**Introduction:**

Misinformation spreading on social media often parallels public crises, such as the outbreak of COVID-19, because people’s behaviors regarding misinformation may be influenced by their typical life circumstances. With the increasing severity of living conditions, misinformation is believed to spread more widely, while corrective behaviors tend to decrease. Furthermore, social comparison also affects the perception of life circumstances and subsequent behaviors. Taking Shanghai’s COVID-19 lockdown as an example, this study examined whether two representative factors—the duration of the lockdown and the satisfaction with relief measures—affected people’s tendency to share and correct misinformation. By employing the third-person effect (TPE) theory, the underlying mechanisms of social comparison were also explored.

**Methods:**

An online survey was conducted in April 2022, when the Zero-COVID policy was implemented in Shanghai. In addition to questions about life circumstances, a third-person perception scale, a behaviors of sharing misinformation scale, and a behaviors of correcting misinformation scale were included in the survey. Finally, 7,962 valid responses were collected.

**Results:**

It was found that both behaviors—sharing and correcting misinformation—were affected by life circumstances but in different ways. The evidence also supported the existence of third-person perception (TPP). It was observed that the relationship between satisfaction with relief measures and sharing behavior was mediated by Third-Person Perception.

**Conclusion:**

This study reveals that the proliferation of misinformation during crises is related to the deterioration of people’s perception of life circumstances. Social comparison often plays a significant role, as was reflected by the TPE.

## 1 Introduction

Misinformation disseminated on social media often parallels public health crises, such as the outbreak of COVID-19 (Cinelli et al., 2020; Fleming, 2020). Unlike the era of traditional media, where professional media outlets played the role of gatekeepers, people’s sharing behavior on social media significantly aggravates the spread of misinformation (Li et al., 2020; Su et al., 2021; Shi et al., 2022). Nevertheless, such aggravations are kept within limits because some people restrain themselves from sharing information that is suspected of being false (Vosoughi et al., 2018). In addition, when discovering that a certain piece of information is false, some people actively post corrective messages in their social spaces, which can also be further spread through social networks (Cheng and Luo, 2020). Therefore, understanding the reasons why individuals share and correct misinformation is an important research topic in the era of social media. As misinformation often leads to great panic during public health crises (Cinelli et al., 2020), it is important to investigate the relevant mechanisms.

Thus far, scholarly responses to such topics have mainly focused on individual factors, such as cognitive biases and socio-affective factors (Ecker et al., 2022). For instance, “status-led” motivations, such as the need for “seeking truth” and “being heard,” are considered the primary driving forces behind people sharing information whose authenticity is in question (Bright, 2016; Duffy et al., 2019; Apuke and Omar, 2021). The third-person effect (TPE) theory proposed by Davison (1983) is often employed in research. Studies based on it argue that the other-self gap of the perceptual media effect (third-person perception, TPP) is positively correlated with social enhancement motivations, which suppress the spread of misinformation (Talwar et al., 2020) and promote corrective behaviors (Koo et al., 2021).

However, few studies have examined how socio-environmental factors influence people’s sharing and correction of misinformation, particularly the relationship between socio-environmental factors and individual factors. This is likely because, in many scenarios, there are too many dimensions of socio-environmental factors related to a given topic, which leads to difficulty in constructing models. For example, in research on the TPE theory, some scholars have often found themselves constrained by the complexity of socio-environmental factors and called for research conducted in appropriate contexts where external factors are more unified (for a systematic review, see Sun et al., 2008a).

In the first half of 2022, a surge in COVID-19 infections broke out in Shanghai, and strict lockdown measures were implemented to curb the situation (Taylor, 2022). On the one hand, almost everyone in Shanghai was restricted to their communities and even required to “stay at home;” on the other hand, to ensure that people’s basic necessities were met, free “relief packages” with food and groceries were delivered by the local governments (Nam et al., 2022; Wang et al., 2022). It is noteworthy that, unlike previous outbreaks, the symptomatic rate, severe rate, and lethal rate in Shanghai were significantly lower (Zhang X. et al., 2022). Thus, people focused more on the inconveniences of daily life brought about by the lockdown, rather than worrying about getting sick or other issues related to the virus itself (Nam et al., 2022; Zhang B. et al., 2022). According to a series of news reports (e.g., Verma, 2022; Yang, 2022) and our observations, the concerns about the two measures mentioned above—the apprehension over the continuation of the lockdown and the satisfaction with the relief package—were the two most mainstream social topics for all citizens during that period. This enabled us to engage these two socio-environmental factors to succinctly reflect people’s life circumstances.

Therefore, we aimed to examine whether and how people’s life circumstances during public crises influence them to share and correct misinformation. In particular, the roles of “status-led” motivations and TPP were investigated. We hope that the engagement of the socio-environmental factors not only helps to better clarify the reasons behind people’s behaviors regarding misinformation but also improves relevant theoretical models such as TPE.

## 2 Literature review

### 2.1 The influence of social circumstances on sharing misinformation

Since uncertainty is a common feature of a public health crisis, deterministic information, such as scientific assessments of a pandemic’s progression and interpretations of the legitimacy of policies, occasionally loses its potency after publication because of the rapid change in social circumstances (Garfin et al., 2020; Gollust et al., 2020). As a result, in terms of information production, some social media users fabricate messages by cobbling together irrelevant, half-true, and even pseudo-scientific information to fill the void caused by the absence of reliable ones (Reyes et al., 2021; Craig and Vijaykumar, 2023; Song et al., 2023). During the sharing process, some people also repost misinformation, either knowingly or unknowingly, to express their skepticism toward authoritative information (Cinelli et al., 2020; Wang and Zhang, 2023) and/or to protect their friends from possible harm (Duffy et al., 2019). In brief, misinformation on social media largely arises due to the uncertainty surrounding public health crises.

Existing studies on “uncertainty” have mainly measured relevant variables in indirect ways and from micro perspectives, although few variables indicating the uncertainty of socio-environmental circumstances have been engaged. For example, Wang and Zhang (2023) focused on the extent to which individuals were informed about the pandemic as an independent variable and found a significant effect of it on the intentions to share misinformation. Although it did reflect individual differences of perceived uncertainty, the subjective factors, such as the ability to obtain true information, did not appear to be entirely controlled. Indeed, in most scenarios, there are likely too many dimensions of socio-environment factors, which makes it difficult to identify succinct and unified variables that represent uncertainty on a macro level.

As mentioned above, Shanghai’s lockdown provided a context where people’s concerns about the pandemic were centered on the inconveniences caused by changes to their normal work and life (Nam et al., 2022; Zhang B. et al., 2022). In addition, according to the “rumor buster” platform proposed by a local state-owned media,^1^ the misinformation that was spread on social media from March to May 2022 was generally related to the lockdown and “relief package.” In other words, as the duration of the lockdown and the relief package for everyday life were two crucial socio-environmental factors, they could indicate, in a unified manner, the uncertainty of that period among different individuals. Specifically, the longer the lockdown lasted,^2^ the greater the uncertainty people experienced, making them more likely to share misinformation. On the contrary, people’s satisfaction with the relief packages might be negatively correlated with their behaviors of sharing misinformation because adequate relief packages could mitigate perceptual uncertainty.

Moreover, one obstacle in researching misinformation is the difficulty of accurately measuring the behavior of sharing misinformation because, in most cases, such as political fake news and business rumors, the time spans involved are likely lengthy, making it difficult for participants to accurately report their relevant behaviors. Researchers thus have to use a scale to measure the intention to share misinformation, which can be too indirect, because it is not easy for individuals to identify whether the messages they have shared are misinformation. Nevertheless, the current study could collect more accurate data by asking participants to recall how many times they shared information that they later realized was not true because during Shanghai’s lockdown, the government and communities frequently released information related to the pandemic, including corrections of misinformation (Chen and Tian, 2024). In addition, the rapid development of the situation could quickly render certain information false, such as whether a certain community was going to be locked down (Nam et al., 2022). Compared with other scenarios, people could relatively easily identify which messages they had shared that might be misinformation. Hence, we used people’s estimation of the number of times they shared misinformation as an indicator of their actual behaviors.

Therefore, we present our hypotheses below:

*H1a*: During Shanghai’s lockdown, the duration of the lockdown (abbreviated to dLD) positively predicts people’s estimation of having shared misinformation (abbreviated to eSM).

*H1b*: During Shanghai’s lockdown, satisfaction with the relief packages (abbreviated to saRP) negatively predicts people’s estimation of having shared misinformation (eSM).

### 2.2 The influence of social circumstances on correcting misinformation

The influences on people’s behavior to correct misinformation can be assumed to move in the opposite direction.

Corrective actions on social media are defined as posting or sharing messages that indicate the faults or inaccuracies of misinformation (Rojas, 2010; Rojas et al., 2016). Regarding the factors that influence people to do so, existing research often examines from the perspectives of media and individuals, such as the type of messages (Chua et al., 2017), individual cognition (Cheng and Luo, 2020), and demographic factors (Craig and Vijaykumar, 2023).

However, it seems that environmental factors also play a role, which has been examined to a limited extent. According to the theory of Reasoned Action and Planned Behavior TRA; (Ajzen, 1985; Ajzen, 2001), people’s actions are often “reasoned” as reactions to their life circumstances, and proactive behaviors, such as altruistic actions, are affected by socio-environmental factors, such as social pressure and social mood. When people perceive less social pressure or a better social mood, they tend to increase prosocial behaviors because they have surplus motivation to engage themselves in altruistic behaviors after feeling satisfied. In addition, a positive social mood leads them to optimistically evaluate the effects of such behaviors. On the contrary, greater social pressure and a poorer social mood negatively influence prosocial behaviors because of the insufficient motivation to engage in altruistic behaviors and a pessimistic assessment of behavioral effects. Applying this theory to the current study, with the deterioration of life experiences or the increase of uncertainty of the future, people’s prosocial behaviors, such as correcting misinformation, are more likely to decline because people may pay more attention to their own issues than to society or they might feel such behaviors cannot have a positive effect in chaotic circumstances.

As mentioned earlier, it is not easy to identify socio-environmental factors that are universally applicable to all individuals because different groups are usually concerned with different dimensions of the social environment. However, in the context of Shanghai’s lockdown, the factors indicating life circumstances were almost unified to a few dimensions, namely the duration of the lockdown and the access to living necessities. This allowed us to incorporate socio-environmental factors into the model of corrective actions and to expand the understanding of TRA in the context of social communication. We thus present the hypotheses below:

*H2a*: During Shanghai’s lockdown, the duration of the lockdown (dLD) negatively predicts the behavior of correcting misinformation (abbreviated to bCM).

*H2b*: During Shanghai’s lockdown, satisfaction with the relief packages (saRP) positively predicts the behavior of correcting misinformation (bCM).

### 2.3 Third-person effects in the spread of misinformation

Behind these possible influences of socio-environmental factors, people’s evaluations of the effect of misinformation are usually considered one of the crucial socio-psychological mechanisms. For instance, Rojas (2010) suggested that correcting actions partially stems from people’s motivation to have “their own views heard.” Arif et al. (2017) found that the “imagined audiences” of individuals vary, which leads them to make different decisions regarding corrective actions. Such evaluations include not only the perceptual importance of a message but also the perceived other-self gap of the media effect. The latter is well known as the third-person effect (TPE), initially proposed by Davison (1983). Essentially, it suggests that the majority of people tend to believe that others are more vulnerable than themselves when confronted with media messages. The extent of such a gap is called Third-Person Perception (TPP). TPP is influenced by individuals’ personalities and external factors, which are known as the “perceptual component” of the TPE. It further leads to different consequences, which are known as the “behavioral component” (for systematic reviews, see Sun et al., 2008a; Eisend, 2017). According to these classic topics of the TPE, studies usually address three research questions: the general pattern of Third-Person Perception, the influencing factors of TPP, and how TPP influences people’s behaviors.

#### 2.3.1 General patterns of TPP

The general other-self gap of the perceptual effect of misinformation has been widely observed, including in the context of the COVID-19 pandemic (e.g., Corbu et al., 2020; Koo et al., 2021; Yang and Tian, 2021; Zhu et al., 2021). These studies found that people often believe that others are more likely to be affected and even deceived by misinformation than they themselves are. Therefore, we aim to provide more empirical evidence by testing the following hypothesis:

*H3*: There is the third-person perception of misinformation during Shanghai’s lockdown for the COVID-19 outbreak.

#### 2.3.2 The influence factors of TPP

Research on the perceptual component of the TPE has focused on exploring the influencing factors of TPP, aiming to discuss the fundamentals of such psychological bias. Individual personality has been identified as one of the major elements. For instance, early research (Meirick, 2002) on the TPE revealed that those with higher self-esteem or self-enhancement demonstrate a greater TPP. Furthermore, some scholars (Gunther, 1991; Park and Salmon, 2005; Tsay-Vogel, 2016) incorporated the social comparison theory (SCT) as another explanation for TPP. They argued that in addition to individual differences, external factors also affect people’s evaluation of media effects. According to the classic assertions of the SCT (Chapin, 2000; Suls et al., 2002; Li, 2008), one of the reasons for cognitive biases is overconfidence and overoptimism. Specifically, it has been found that TPP is significantly correlated with people’s optimistic bias (Chapin, 2000), particularly when comparisons with others are involved (Li, 2008). Regarding the reasons behind it, scholars have further argued that with the decrease in optimism, feelings of overconfidence and superiority in comparison to others diminish, which leads people to consider the similarity between themselves and others, fostering empathy. Furthermore, such an optimistic bias is found to be related to individual factors such as personal skills (Li, 2008) and individual patterns of media use (van der Meer et al., 2022).

However, few studies have been conducted from the perspective of the influence of socio-environmental factors on TPP. As Park and Salmon (2005) noted, one of the difficulties in research design is the fact that “there are too many different kinds of topics and issues,” which makes it challenging to identify and measure external factors because of non-uniform dimensions of social issues. If the correlation between socio-environmental factors and TPP is significant, the TPE can be considered operating at the social level rather than solely being driven by individual personality. The two aforementioned succinct variables during Shanghai’s lockdown, namely the duration of the lockdown and satisfaction with the relief packages, were unified among individuals and reflected the negative and positive social circumstances, respectively. This allowed us to explore the relationship between these factors and TPP more directly.

By incorporating the aforementioned research on the relationship between optimism and TPP (Chapin, 2000; Li, 2008) into the current study, it was assumed that when an individual experiences a longer duration of lockdown, his or her confidence in the certainty of future life may decrease. For instance, as more information about the pandemic, such as rational predictions about the time of unlocking, was later confirmed to be inaccurate, individuals’ confidence was severely affected. Then, he or she was more likely to consider that the future life would exceed his or her judgment, and that his or her ability to judge the authenticity of information was not superior to others. The gap between the influence of misinformation on him/herself and on others (TPP) declined. Similarly, satisfaction with the relief packages was often the result of comparisons with others on social media [e.g., comparing one’s situation to others’ posts, Nam et al. (2022)]. Since the relief packages were usually delivered by communities, when people were more satisfied with the relief packages, they were more optimistic and more confident in the community where they lived. With the increase in such satisfaction, they likely perceived themselves as less influenced by misinformation about the lockdown because much misinformation was about doubts regarding the relief policies. Therefore, we propose the following hypotheses:

*H4a*: The duration of the lockdown (dLD) negatively predicts the TPP of misinformation (abbreviated to TPPEM).

*H4b*: Satisfaction with the relief packages (saRP) positively predicts the TPP of misinformation (TPPEM).

#### 2.3.3 The consequences of TPP

Regarding the behavioral component of the TPE, a large number of studies (e.g., Nathanson et al., 2002; Sun et al., 2008b; Zhu et al., 2021) have demonstrated positive correlations between TPP and altruistic behaviors, as well as negative associations between TPP and harmful behaviors. In the case of misinformation, Yang and Horning (2020) found that those who have a greater TPP of fake news are less likely “intended” to spread it. Similarly, existing research (Rojas, 2010; Koo et al., 2021) on corrective actions revealed that TPP positively predicted the “intention” to correct. As the behaviors of sharing and correcting misinformation were measured more precisely and directly in the current study, we aim to use a more objective factor as the dependent variable to test the following hypothesis:

*H5*: The TPP of misinformation (TPPEM) negatively predicts people’s estimation of having shared misinformation (eSM).

*H6*: The TPP of misinformation (TPPEM) positively predicts the behavior of correcting misinformation (bCM).

### 2.4 The mediation effect of TPP

As TPP was likely affected by the social circumstances during Shanghai’s lockdown and also possibly influenced people’s behaviors, it was reasonable to further assume that TPP played a mediation role. Nevertheless, from the statistical perspective (Hayes, 2021), even if the former two hypotheses, namely H4 and H5&6, were both valid, the mediation effect did not necessarily occur. Theoretically, in previous research on social comparison, there were different findings regarding whether the results of comparing social factors are transmitted to behaviors (Gerber et al., 2018). It may reflect the subtle mechanism of a given scenario. In the context of the current study, if TPP is correlated with both the independent variable (e.g., duration of lockdown) and the dependent variable (e.g., people’s estimation of having shared misinformation), but the mediation effect of it is not significant, we could argue that there is no sufficient evidence supporting that people conduct social comparisons when they consider their behaviors related to misinformation. Hence, we aim to investigate the mediation effects on the behaviors of sharing (RQ1) and correcting (RQ2) misinformation:

*RQ1a*: Does the TPP of misinformation (TPPEM) mediate the influence of the duration of the lockdown (dLD) on people’s estimation of having shared misinformation (eSM);

*RQ1b*: Does the TPP of misinformation (TPPEM) mediate the influence of satisfaction with the relief packages (saRP) on people’s estimation of having shared misinformation (eSM)?

*RQ2a*: Does the TPP of misinformation (TPPEM) mediate the influence of the duration of the lockdown (dLD) on the behavior of correcting misinformation (bCM);

*RQ2b*: Does the TPP of misinformation (TPPEM) mediate the influence of satisfaction with the relief packages (saRP) on the behavior of correcting misinformation (bCM)?

### 2.5 The role of the overall perceptual effect of misinformation

Research on the TPE (Schmierbach et al., 2008; Sun et al., 2008b; Rojas, 2010) generally suggests that the overall perceptual media effect or the perceived total influence of certain content is an important variable, which is calculated by summing the perceptual effect on others and on oneself because it always plays its role in conjunction with TPP. Regarding the scenarios of misinformation, people who perceive media or content as influential are considered more likely to take actions, including both sharing misinformation (Duffy et al., 2019; Greifeneder et al., 2020; Apuke and Omar, 2021) and correcting it (Rojas, 2010). Indeed, from the perspective of social morality, it seems to be harmful to share misinformation that is perceived as influential. However, as Duffy et al. (2019) noted, when faced with a specific message, people struggle to distinguish between true and false information and often share it for the purpose of verifying its authenticity. Thus, the overall perceptual effect of misinformation implies both the need to answer one’s questions and the satisfaction derived from being heard by others.

Moreover, similar to TPP, such overall perception is likely influenced by social circumstances. During Shanghai’s lockdown, when the inconvenience of everyday life and the uncertainty of the circumstances increased, the perceived effect of misinformation may have been amplified.

Therefore, to ensure the effectiveness of our models, this study incorporated the overall perceptual effect of misinformation as another potential mediation variable, proposing the following hypotheses and research questions:

*H7*: (a) The duration of lockdown (dLD) positively predicts the overall perceptual effect of misinformation (abbreviated to allEM), whereas (b) satisfaction with the relief packages (saRP) negatively predicts it.

*H8*: The overall perceptual effect of misinformation (allEM) positively predicts people’s estimation of having shared misinformation (eSM).

*H9*: The overall perceptual effect of misinformation (allEM) positively predicts the behavior of correcting misinformation (bCM).

*RQ3*: Does the overall perceptual effect of misinformation (allEM) mediate (a) the influence of the duration of the lockdown (dLD) and (b) satisfaction with the relief packages (saRP) on people’s estimation of having shared misinformation (eSM);

*RQ4*: Does the overall perceptual effect of misinformation (allEM) mediate (a) the influence of the duration of the lockdown (dLD) and (b) satisfaction with the relief packages (saRP) on the behavior of correcting misinformation (bCM)?

**Figure 1:**
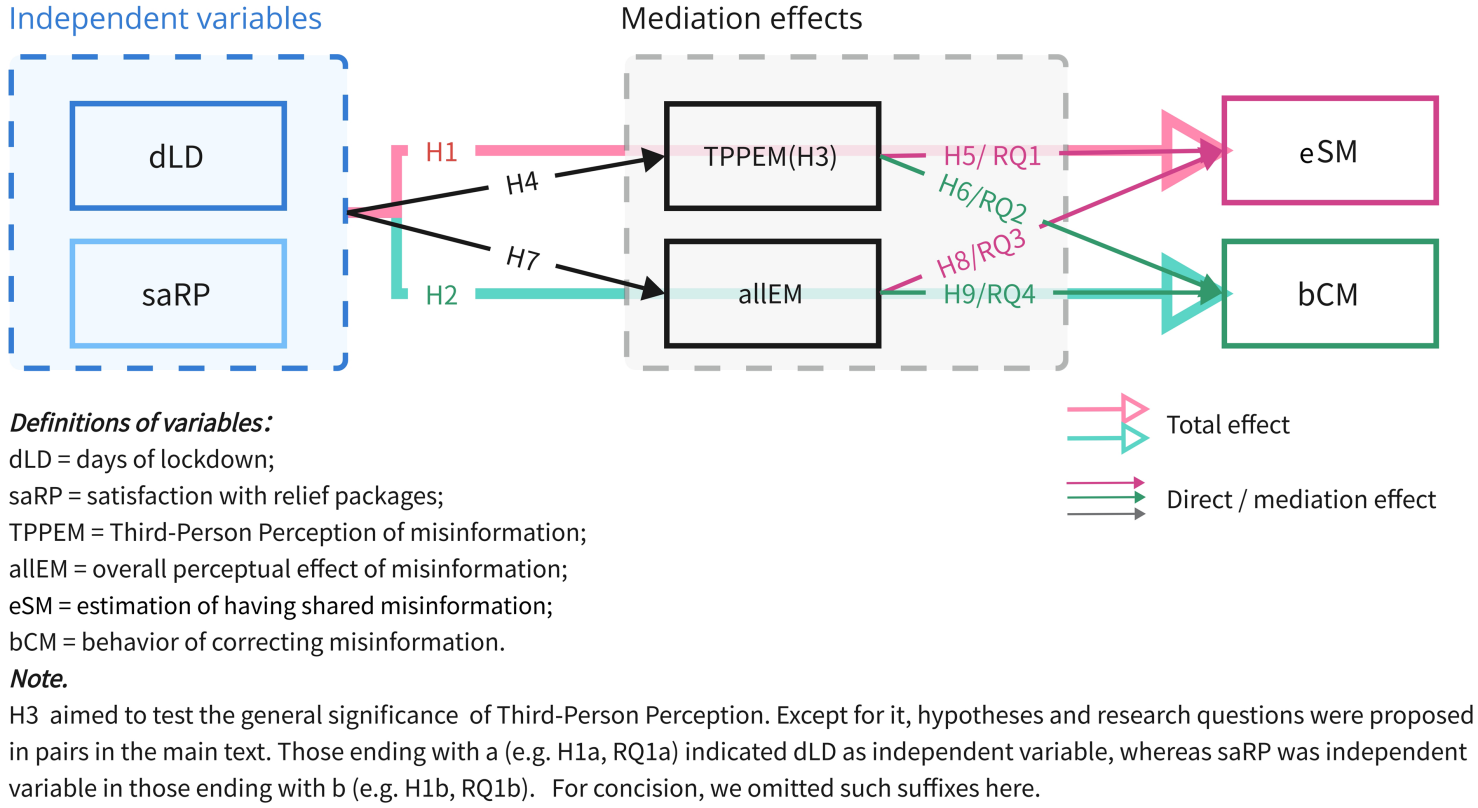
Research models.

In summary, we proposed two parallel models addressing the behaviors of sharing and correcting misinformation, respectively. The influencing factors on TPP were equivalent. However, the influence paths, such as the overall effect of life circumstances and the mediating effects of TPP, might have differed between both models. The complete models for all hypotheses and research questions are illustrated in Figure 1.

## 3 Methods

This study used an online survey method. Snowball sampling was employed since the questionnaire distribution relied on social network dissemination. The self-reported online survey was conducted from the 7th to 9th of April 2022, using Tencent Survey. Data analyses were performed using SPSS 24 and JASP 0.16.4.

### 3.1 Sampling

Owing to the word-of-mouth strategy^3^ used for recruiting participants, nearly 10,000 questionnaires were collected. Attention and eligibility checks were incorporated into the questionnaire using the methods proposed by Meade and Craig (2012). Common sense-based questions and questions whose answers could be quickly found on the same page were both included. Any incorrect responses to these questions resulted in those cases being marked as invalid. As recommended by Johnson (2005), we also eliminated any responses that had the same choices for all odd-numbered or all even-numbered questions. Finally, more than 2,000 responses were excluded, and 7,962 participants were included for further analysis. These 7,962 participants represented 210 of 213 towns and sub-districts in Shanghai. Their ages ranged between 13 and 85 (*M* = 38.153, SD = 11.875). A total of 51.2% of the participants were female (*n* = 3,885), while 48.8% were male (*n* = 4,077). A total of 68.4% of them (*n* = 5,448) held a Bachelor’s degree or higher (for demographic statistics, see Table 1). Their demographic composition was similar to that of the population census in 2020 in terms of sex, education level, income, and the districts in which they lived.

**Table 1:** Descriptive statistics and reliability test.

Variable	Count (%)	Mean	SD	Cronbach’s *α* (items)
Sex				
Male	3,885 (48.8%)			
Female	4,077 (51.28%)			
Age		38.15	11.88	
Education				
Junior middle school or less	239 (3.08%)			
Senior middle school	844 (10.68%)			
College	1,431 (18.0%)			
Bachelor’s degree	3,832 (48.1%)			
Master’s degree or above	1,616 (20.3%)			
Monthly income (RMB)				
<=3,000	325 (4.0%)			
3,001 ~ 10,000	2,792 (35.0%)			
10,001 ~ 20,000	1847 (23.2%)			
>20,000	1,522 (19.1%)			
No answer	1,476 (18.5%)			
dLD		16.07	6.971	
saRP		2.972	1.131	0.821 (6)
Perceptual effect on SELF		3.664	1.607	0.787 (3)
Perceptual effect on OTHERS		5.03	1.497	0.924 (3)
eSM		2.822	3.968	
bCM		3.808	1.96	

*N = 7,962.*

### 3.2 Measurement for dependent variables

As emphasized earlier, one of the purposes of this study was to use more accurate behavioral counts as dependent variables, rather than indirectly measuring intentions, willingness, or likelihood. Thus, the participants were asked to recall and count their behaviors of sharing and correcting misinformation as follows: Please recall and estimate how many times you shared messages that were later confirmed misinformation during this wave of the COVID-19 outbreak in Shanghai (from 1st March 2022 onward),^4^ and how many times you created or shared messages aimed at correcting certain misinformation (e.g., your results from fact-checking rumors or misinformation-refuting messages published by official media). Each question consisted of four sub-questions, indicating the four most frequently used mainstream social network channels among Chinese people (Stockmann and Luo, 2017; Fu and Cook, 2019; Shen and Gong, 2019). The participants were asked to specify the counts of the above behaviors in one-to-one chats, in group chats, on private social media (e.g., WeChat Moments), and on public social media (e.g., Weibo). In addition, we provided a calculation prompt below these questions to assist the participants whose behaviors were too frequent, such as “sharing every day = 35–40” and “sharing 3–5 times a week = 16–27.” The final values for both behaviors were calculated as the average of the four items. For people’s estimation of having shared misinformation (eSM), the results were *M* = 2.822, SD = 3.968; whereas for the behavior of correcting misinformation (bCM), the results were *M* = 3.808, SD = 1.960.

### 3.3 Measurement for independent variables and mediation variables

#### 3.3.1 Variables indicating the life circumstances

The duration of the lockdown (dLD) was indicated with an exact value. The participants were asked to report the number of days (*M* = 16.070, SD = 6.971) their communities had been under the lockdown.

Unlike relief packages delivered during other natural disasters, which typically include emergency items such as instant noodles, the relief packages during Shanghai’s lockdown included a large amount of daily food and household items, such as vegetables, meat, and cleaning supplies. Therefore, existing research did not provide mature scales for measuring this variable, and we had to create an original scale to measure satisfaction with the relief packages (saRP).

A focus group interview was organized with 15 experts in journalism, psychology, and sociology, who were invited to participate in a WeChat group chat for discussion. All of the experts were from Shanghai, had experienced the lockdown, and had received the relief packages. After the discussion, they suggested that diversity, quality, and suitability were the main aspects that concerned the people the most regarding their satisfaction with the relief packages. We created a 5-point Likert scale with six items, three of which were inversed, including “Do you agree that the foods and groceries in the relief packages are: diversified/sufficient/able to meet my needs; of poor quality/imbalanced in collocation/unsuited to me” (1 = strongly disagree; 5 = strongly agree). The participants could also choose the option “I have not received any relief packages” alternatively. For the 7,602 participants who had received the relief packages, the value of saRP was calculated by averaging the six items after re-coding the latter three (six items, *M* = 3.113, SD = 0.949, *α* = 0.821). A total of 360 participants claimed they had not received any relief packages, their values were set to 0. Combining both situations, the overall descriptive statistics of saRP were *M* = 2.972 and SD = 1.131.

#### 3.3.2 Perceptual media effects of misinformation

As both TPP and the overall effect of misinformation are calculated based on the perceptual effects on oneself and on others (Schmierbach et al., 2008; Sun et al., 2008b; Rojas, 2010), the participants were asked to evaluate these two effects by answering two parallel groups of questions. Specifically, a 7-point Likert scale with three items proposed by Yang and Tian (2021) was used in each group. The items included the following: “misinformation regarding COVID-19 attracted MY/OTHERS’ attention,” “the content of misinformation regarding COVID-19 had been persuasive to ME/OTHERS,” and “misinformation influenced MY/OTHERS’ decisions regarding COVID-19.” The average value of the items regarding “my” or “me” indicated the perceptual effect on oneself (three items, *M* = 3.664, SD = 1.607, *α* = 0.787), while the average value of “others” indicated the perceptual effect on others (three items, *M* = 5.030, SD = 1.497, *α* = 0.924). The TPP of misinformation (TPPEM) was calculated as the difference between the two values, subtracting the former from the latter (*M* = 1.365, SD = 1.510); whereas the overall effect of misinformation (allEM) was the sum of both values (*M* = 8.693, SD = 2.714).

### 3.4 Analytical procedure

First, to examine the existence of the Third-Person Perception of misinformation (H3), a paired *t*-test was conducted.

Second, since the results of structural equation modeling (SEM) include a series of linear regressions between each pair of variables in the influence path (Hayes, 2021), two SEM were conducted to investigate the models of sharing and correcting misinformation. Rosseel (2012)’s software, built-in JASP 0.16.4., was used for the analysis. Specifically, H1 and H2 were tested using the total effects of the models, the results of H4 ~ H9 were derived from paired correlations, and RQ1 ~ RQ4 were assessed through mediation effects.

Demographic variables (i.e., sex, age, education, and income) were controlled in all analyses because previous research on similar scenarios indicated that they were related to sharing behaviors during COVID-19 and third-person effects (e.g., Sun et al., 2008a; Wang and Zhang, 2023). Some participants (*n* = 1,476, 18.5%) did not report their income. These missing values were replaced with the mean.

## 4 Results

### 4.1 The overall influence of social circumstances

H1a, the duration of lockdown (dLD) positively predicts people’s estimation of having shared misinformation (*β* = 0.036, *p* = 0.001), and H1b, satisfaction with the relief packages (saRP) negatively predicts people’s estimation of having shared misinformation (*β* = −0.076, *p* < 0.001), were both supported.

H2 predicted a positive correlation between life circumstances and the behavior of correcting misinformation (bCM), which was partially supported. On the one hand, neither the total effect (*p* = 0.904) nor the direct effect (*p* = 0.732) of the duration of lockdown (dLD) on bCM was found to be significant. Thus, H2a was not supported. On the other hand, the total effect of saRP was found to be significant (*β* = 0.089, *p* < 0.001). Thus, H2b was supported.

### 4.2 The overall pattern of the third-person perception of misinformation

**Figure 2:**
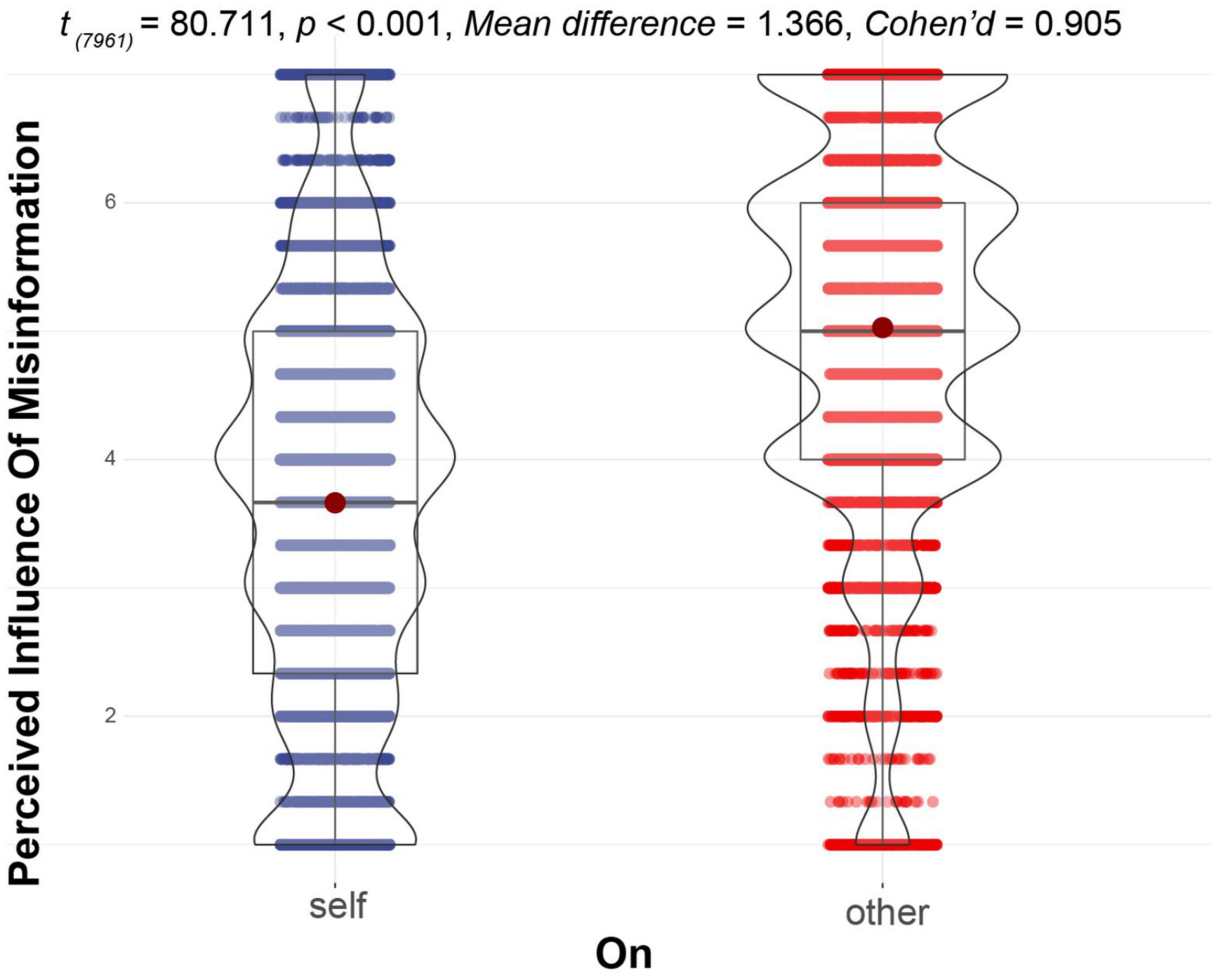
The overall pattern of the third-person perception of misinformation.

H3 hypothesized the existence of TPP. The result of a paired *t*-test (see Figure 2) showed that the perceptual effect of misinformation on others was significantly greater than that on oneself (*t*_7961_ = 80.711, *p* < 0.001, Cohen’d = 0.905). On average, the participants scored the effect of misinformation on others 1.365 points more than that on themselves. Thus, H3 was strongly supported.

### 4.3 The results of the SEM

#### 4.3.1 How social circumstance influenced the perceptual media effect of misinformation

H4 hypothesized a negative correlation between the duration of lockdown (dLD) and the TPP of misinformation (TPPEM) and a positive correlation between satisfaction with the relief packages (saRP) and TPPEM. For the former (H4a), no significant result was found (*p* = 0.609), whereas a significant result was observed for the latter (*β* = 0.039, *p* < 0.001), thereby supporting H4b.

H7 hypothesized similar correlations between the independent variables and the overall perceptual effect of misinformation (allEM). As expected, a positive correlation was found between dLD and allEM (*β* = 0.043, *p* < 0.001), while a negative correlation was found between saRP and allEM (*β* = −0.043, *p* < 0.001). Thus, H7a and b were supported.

#### 4.3.2 The influence path on people’s estimation of having shared misinformation

**Figure 3:**
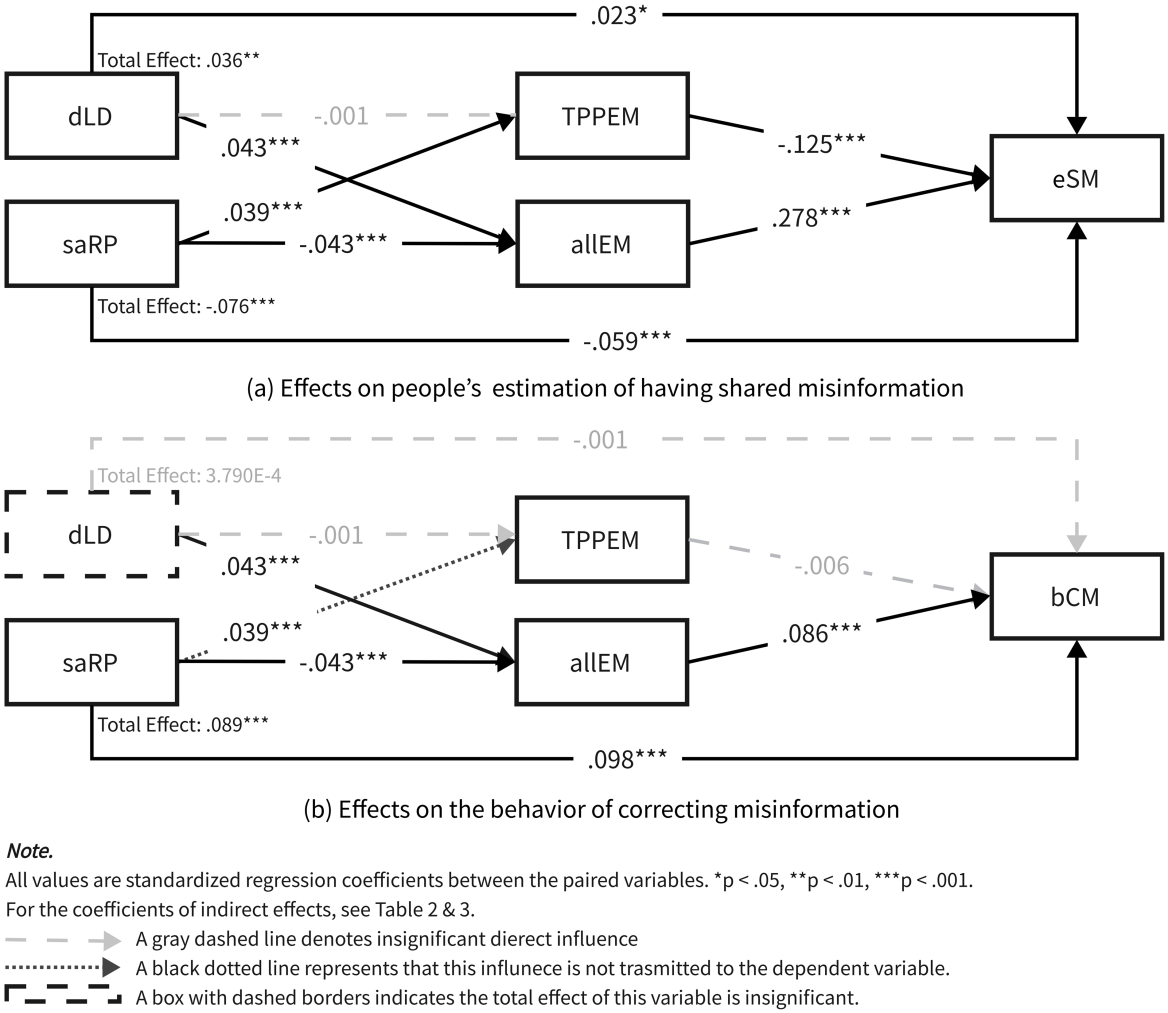
The results of the SEM.

As both H1a and H1b were supported, the model regarding the people’s estimation of having shared misinformation (eSM), namely, H5 and RQ1, as well as H8 and RQ3, were further investigated. The summary of the results is illustrated in Figure 3a and Table 2. According to the total adjusted *R*^2^, 11.6% of the variation in eSM can be explained by this model.

**Table 2:** Effects on the behavior of sharing misinformation.

IV	Path			DV	Estimate	SE	*Z*	*p*	95% CI	
									Lower	Upper
dLD		→		eSM	0.023^*^	0.011	2.171	0.030	0.002	0.044
dLD	→	TPPEM	→	eSM	7.124E-4	0.001	0.512	0.609	−0.002	0.003
dLD	→	allEM	→	eSM	0.012	0.003	3.934	<0.001	0.006	0.018
**dLD**	**Total effects**			**eSM**	**0.036** ^**^	**0.011**	**3.207**	**0.001**	**0.014**	**0.058**
saRP		→		eSM	−0.059^***^	0.011	−5.486	<0.001	−0.080	−0.038
saRP	→	TPPEM	→	eSM	−0.005^***^	0.001	−3.343	0.009	−0.008	−0.002
saRP	→	allEM	→	eSM	−0.012^***^	0.003	−3.893	0.008	−0.018	−0.006
**saRP**	**Total effects**			**eSM**	**−0.076** ^***^	**0.012**	**−6.724**	**<0.001**	**−0.098**	**−0.054**

Total *R*^2^ of eSM = 11.6%. ^*^p < 0.05, ^**^p < 0.01, and ^***^p < 0.001.

H5 hypothesized a negative influence of the TPP of misinformation (TPPEM) on people’s estimation of having shared misinformation (eSM). H8 predicted a positive influence of the overall perceptual effect of misinformation (allEM) on eSM. As expected, TPPEM was found to negatively predict eSM (*β* = −0.125, *p* < 0.001), thereby supporting H5. In addition, allEM positively predicted eSM (*β* = 0.278, *p* < 0.001), providing evidence for H8.

Regarding the mediation effects that RQ1 and RQ3 focused on, three significant paths were identified. It was not surprising that the influence of dLD was mediated by allEM (*β* = 0.012, *p* < 0.001) but not by TPPEM (*p* = 0.069) because the correlation between dLD and TPPEM was not significant, as mentioned earlier. This was one mediation effect in this model. The other two involved the influence of saRP, which was mediated by both TPPEM and allEM. In addition, there were direct effects in both influence paths of dLD and saRP, suggesting that there were some mechanisms that cannot be explained by the third-person effect.

#### 4.3.3 The influence path on the behavior of correcting misinformation

Similarly, H6 and RQ2, as well as H9 and RQ4, were investigated in the model regarding the behavior of correcting misinformation (bCM). Figure 3b and Table 3 demonstrate the results. The total adjusted R^2^ of this model was 2.5%.

**Table 3:** Effects on the behavior of correcting misinformation.

IV	Path			DV	Estimate	SE	*Z*	*p*	95% CI	
									Lower	Upper
dLD		→		bCM	−0.001	0.003	−0.342	0.732	−0.007	0.005
dLD	→	TPPEM	→	bCM	7.890E-6	2.377E-5	0.332	0.740	−3.869E-5	5.447E-5
dLD	→	allEM	→	bCM	0.001^***^	3.889E-4	3.713	<0.001	6.818E-4	0.002
**dLD**	**Total effects**			**bCM**	**3.790E-4**	**0.003**	**0.120**	**0.904**	**−0.006**	**0.007**
saRP		→		bCM	0.098^***^	0.020	5.013	< 0.001	−0.060	−0.136
saRP	→	TPPEM	→	bCM	3.353E-4	7.749E-4	−0.433	0.665	−0.002	0.001
saRP	→	allEM	→	bCM	−0.009^***^	0.002	−3.678	<0.001	−0.014	−0.004
**saRP**	**Total effects**			**bCM**	**0.089** ^***^	**0.020**	**4.517**	**<0.001**	**−0.050**	**0.127**

Total *R*^2^ of bCM = 2.5%. ^*^p < 0.05, ^**^p < 0.01, and ^***^p < 0.001.

H6 hypothesized that the TPP of misinformation positively predicts bCM; however, no significant evidence was found (*p* = 0.663). As a result, the mediation effect assumed in RQ2 could not be observed either.

H9 hypothesized that the overall perceptual effect of misinformation (allEM) positively predicts the behavior of correcting misinformation (bCM), and this was found to be significant (*β* = 0.089, *p* < 0.001). The mediation effects of it, as addressed in RQ4, were also observed in both influence paths of dLD (*β* = 0.001, *p* < 0.001) and saRP (*β* = −0.009, *p* < 0.001). For the former, since H2a was not supported, such mediation effects may have little theoretical meaning. For the latter, the direction of the total effect of saRP on bCM was opposite to that of the mediation effect, suggesting a relatively complex mechanism. As H2b and H7b were both supported and the mediation effect of allEM on bCM was significant, the direct effect of saRP was hedged by the mediation effect. As satisfaction with the relief measures increased, people’s corrective actions generally grew. This may be because of reduced social pressure or an improved social mood, as proposed in Section 2.2. Meanwhile, the increase in saRP also led to a decline in allEM, which made people consider it less necessary to engage in correcting behaviors. However, the effect of the mediation was so small that the total effect was dominated by the direct effect.

## 5 Discussion

### 5.1 Different mechanisms of the influence of environmental factors on behaviors

Generally, the spread of misinformation on social media was whetted by factors representing unfavorable life circumstances, whether they were restricting measures that caused inconvenience (the duration of lockdown, proposed in H1a) or dissatisfaction with relief measures (the satisfaction with relief packages, proposed in H1b). In addition, the overall perceptual effect of misinformation played mediating roles in both models. These findings support the assumption that perceptual uncertainty caused by negative life circumstances likely led people to seek truth by sharing potential misinformation, which was expected to relieve their confusion and anxiety.

However, people’s estimation of having shared misinformation was more influenced by environmental factors than their corrective actions. The corrective actions appeared to be affected by the satisfaction with the relief package and not by the duration of the lockdown. This could likely be explained from the perspective of the efficiency of a behavior. The theory of reasoned action and planned behavior, as mentioned above, was used to hypothesize that prosocial activities such as correcting misinformation on social media would decline with a deterioration in social pressure and social mood. Here, “efficiency” can be considered as the extent to which such deterioration can be alleviated. The reason why the influence of the duration of the lockdown on correcting behaviors was not supported may be that the variance in the duration of the lockdown could not reflect such social mood. The length of time an individual is restricted inside his/her community certainly reflects the inconvenience that he/she has experienced; however, his/her expectations regarding the social atmosphere generally depend on the macro-level development of the pandemic. Even if a lockdown in a certain community is not lengthy, individuals who live there may still consider the upcoming unfavourability spreading from other communities that have been locked down for longer periods. Consequently, participation in corrective actions may not relieve such anxiety. In contrast, satisfaction with relief packages offers a stable expectation. As scholars (Nam et al., 2022; Wang et al., 2022) observed, people’s satisfaction reflects their confidence in the services provided by the community in which they live. Thus, with the increase in satisfaction, the motivation of an individual to maintain a positive climate of opinions grows for supporting the community he/she lives in. In summary, we argue that only environmental factors with relatively stable development can influence the behavior of correcting misinformation, while those associated with erratic expectations likely cannot.

In addition, a marginal effect was observed in which the overall perceptual effect of misinformation mediated the influence of satisfaction with the relief packages on corrective actions in an opposite direction. However, this influence path cannot fully hedge the direct influence of satisfaction with relief packages. In other words, a very small group of people who were more satisfied with the relief packages might have paid less attention to misinformation and engaged in fewer corrective actions. However, this did not alter the main trend.

### 5.2 The existence of TPP and the mechanisms of the TPE

The current study provides evidence supporting the main assumptions of the third-person effect; however, some findings were inconsistent with previous research, which likely suggests the subtle mechanisms of the TPE in a typical context.

Firstly, the test for the existence of the Third-Person Perception yielded significant results once again.

Secondly, while the positive influence of satisfaction with relief packages on the TPP of misinformation was supported, the negative influence of the duration of the lockdown was not. We would also like to suggest that life circumstances should be considered an influencing factor on TPP. The reason why the duration of the lockdown did not significantly influence TPP is likely similar to the reasoning discussed in Section 5.1. When people in Shanghai compared the media effects of misinformation, they only considered stable social environmental factors. As discussed above, people likely focused less on the lockdown that had already been implemented and more on the uncertainty of how long it might last.

Thirdly, the TPP of misinformation (TPPEM) negatively predicted people’s estimation of having shared misinformation, which can be regarded as a form of altruistic behavior. Furthermore, it mediated the influence of satisfaction with relief packages on the behavior of sharing. We thus argue that the perceptual gap of the effect of misinformation operates at a social level rather than merely at an individual level. In addition, it explains why life circumstances affect such a behavior. Specifically, besides maintaining the opinion climate of communities, the reduction in the spread of misinformation also stems from the motivation to protect others, which can be promoted by social comparison based on positive life circumstances.

Lastly, inconsistent with some prior research (Rojas, 2010; Koo et al., 2021), neither the influence of TPP on the behavior of correcting misinformation was significant, nor was its mediation effect. We would like to discuss such inconsistencies below. Instead of the “intention” or “likelihood” used in these previous studies, we employed the exact count of corrective actions as the dependent variable. The “cost” in reality may be a key distinction between actual conduct and self-reported intentions. According to Mehrabian and Russell's (1974) Stimulus-Organism-Response (SOR) theory, the organic perception related to external stimuli, such as TPP influenced by life circumstances, does not necessarily further lead to responses, such as certain actions regarding misinformation. Instead, it depends on the advantage of gains over efforts. There is hardly any cost when an individual expresses altruistic intentions, while the positive psychological feedback it brings, such as self-affirmation, is evident. Even exercising self-control to reduce harmful behaviors, such as ceasing to spread misinformation, does not incur significant expense. However, effective corrective actions usually call for a strong conviction to fight against fallacies, communication skills to persuade others, and trivial works to identify the authenticity of information (Cotter et al., 2022; Ecker et al., 2022). All of these entail considerable endeavors involving long-term accumulated media literacy and short-term efforts. Therefore, caring for vulnerable others was probably less cost-effective than being heard during Shanghai’s lockdown because the latter only required the action of posting without the pursuit of its persuasiveness. This might be the reason why only the overall perceptual effect of misinformation mediated the influence on the behavior of correcting. In summary, as a consequent behavior of the third-person effect, the variance in sharing misinformation may reflect people’s social comparison, likely due to its relatively low cost. However, correcting misinformation incurs greater costs, and this is why the mediation effect of TPP was not significant.

## 6 Conclusion, implications, and limitations

### 6.1 Conclusion

Owing to the typical research scenarios of Shanghai’s lockdown in 2022 and the relatively large sample size, this study not only provides empirical evidence that life circumstances are one of the factors influencing people’s behaviors regarding misinformation but also explores some subtle mechanisms behind such relationships. “Status-led” motivation played mediating roles in both behaviors of sharing and correcting misinformation; however, Third-Person Perception played a mediating role only in the scenario of sharing.

### 6.2 Implications

Theoretically, the findings of this study imply a more complex mechanism of misinformation-related issues and the third-person effect. People’s responses to misinformation, as well as both the perceptual component and behavioral component of the TPE, should be viewed as “reasoned actions” because they are all related to external factors and are not merely influenced by internal factors, such as personality, emotion, and demographic factors.

In practice, we argue that timely implementation of relief policies during a crisis can help curb the spread of misinformation and encourage people to correct it. Furthermore, as the third-person effect does not play a mediation role, when people correct misinformation, they might not compare themselves with others. Therefore, efforts to encourage individuals to be more proactive in combating misinformation through differentiated relief policies may have limited effectiveness. Administrators only need to indiscriminately increase people’s satisfaction with relief policies.

### 6.3 Limitations

Indeed, although we introduced the TRA (the theory of reasoned action and planned behavior) to propose some of our hypotheses and to explain the main findings, the latent variables of the TRA had not been completely incorporated, especially into the investigation of the TPE. Hence, some conclusions regarding the subtle mechanisms are inferences based on inadequate evidence. This might be the main limitation of the presumed model this study illustrates. We thus suggest that future research employ additional variables, such as the anticipated inconvenience of life, the cost of correcting misinformation, and the perceptual effects of corrective actions, to examine our inferences by combining the theories of the TPE and TRA.

Another limitation of this study is that a completely consistent context is difficult to reproduce because the lockdown of a big city is a rare occurrence. Nevertheless, the model proposed in the current study can likely be applied to other similar scenarios. In our research plan, scenarios that are also highly related to governments’ actions, such as economic downturns and declines in real estate valuations, are currently being explored. For example, regarding misinformation related to small business operations, the behavior of business owners in sharing or correcting misinformation may also be related to governments’ policies. Thus, we hope that more scholars can introduce more similar scenarios to improve the universality of this model. In addition, we encourage research that examines contexts outside of China.

Moreover, there are some inherent limitations to the survey method. First, the variable eSM (behaviors of sharing misinformation) might be underestimated. When a participant did not receive corresponding refutation information, they might not have realized that a previously shared message was false. Nevertheless, such an inaccuracy was consistent for all participants. Thus, while it is highly likely that it did not affect the trend conclusions of the statistical analyses, it might have led to inaccuracies in the detailed patterns, such as regression coefficients. Future research can introduce big data methods to automatically capture participants’ actions on social media to obtain more accurate data and more subtle patterns. Second, the measurement of satisfaction with the relief packages inevitably entailed subjective factors, such as optimism, which might have also influenced TPP. Future research should involve variables related to relevant personalities and control them.

When we finally write the manuscript, the zero-COVID policy in China is abruptly abandoned and people are encountering an unprecedented wave of infection. At this moment, a surge of misinformation is spread on social media again regarding the opening up, the infection, and future policies. This likely implies that research on the correlation between environmental factors and people’s behaviors related to misinformation holds its typical value in the context of contemporary China since the government often implements strong policies and many behaviors on Chinese social media are closely related to the social environment influenced, even shaped, by these policies.

## Data Availability

The datasets presented in this article are not readily available because of anonymity concerns. Readers interested in the data can contact the corresponding author upon reasonable request. Requests to access the datasets should be directed to emy_xiang@sjtu.edu.cn.

## Footnotes

^1^“Rumor buster” was a misinformation-refuting platform proposed by Shanghai United Media Group, a state-owned media. It concluded the widespread misinformation monthly. For example, the top seven pieces of misinformation in April 2022 were listed in https://mp.weixin.qq.com/s/uMYcZCgf6aqBbhQLaXW7lg (Chinese version only), most of which were about the lockdown and relief packages.

^2^It is widely known that almost all communities in Shanghai were locked down equivalently for more than 2 months during this wave of COVID-19 outbreak. However, before 12th April, such undifferentiated policies had not yet been implemented. Till the survey of the current study was conducted, the actions (e.g., lockdown and relief packages) taken by local administrations (e.g., governments of towns and districts) for different communities varied. Thus, sufficient variance of the duration of lockdown was obtained.

^3^In China, the function of “long press the image to scan QR code” is widely employed by mainstream social media software (e.g., WeChat). When people want to share certain content with friends, they are accustomed to sending posters with QR codes, in which there are hyperlinks toward certain content like web pages. Compared to text links, posters with QR codes are often more attractive and motivate more sharing, since they vividly provide preliminary information related to the content. Therefore, on Chinese social media, one way to promote word-of-mouth communication is to design attractive posters and guide the audience to scan the QR code, as well as to share the posters with friends. During the outbreak of COVID-19 in Shanghai, citizens were highly concerned by governments’ policies published at daily press conferences. We designed a series of posters, on which these policies were graphically illustrated (e.g., a flowchart that explained “under what circumstances would a community be locked down”). Such that, people were very willing to share them. On these posters, we also placed a QR code which was linked to our questionnaire. With the widespread dissemination of the posters, we obtained a large number of participants.

^4^The first case of infection during this wave of COVID-19 outbreak in Shanghai was reported at the press conference of Shanghai Municipal Government on March 1st 2022. Hence, we emphasized the date to ensure a unified understanding among all participants. For more information, see https://en.wikipedia.org/wiki/2022_Shanghai_COVID-19_outbreak.

## References

[ref1] Ajzen I. (1985). “From intentions to actions: a theory of planned behavior” in Action control: from cognition to behavior. eds. Kuhl J. Beckmann J. (Berlin, Heidelberg: Springer Berlin Heidelberg), 11–39.

[ref2] Ajzen I. (2001). Nature and operation of attitudes. Annu. Rev. Psychol. 52, 27–58. doi: 10.1146/annurev.psych.52.1.27 11148298

[ref3] Apuke O. D. Omar B. (2021). Fake news and COVID-19: modelling the predictors of fake news sharing among social media users. Telemat Inform 56:101475. doi: 10.1016/j.tele.2020.101475, PMID: 34887612 PMC7390799

[ref4] Arif A. Robinson J. J. Stanek S. A. Fichet E. S. Townsend P. Worku Z.. (2017). “A closer look at the self-correcting crowd: Examining corrections in online rumors” in Proceedings of the 2017 ACM conference on computer supported cooperative work and social computing (Portland, Oregon: Association for Computing Machinery).

[ref5] Bright J. (2016). The social news gap: how news Reading and news sharing diverge. J. Commun. 66, 343–365. doi: 10.1111/jcom.12232

[ref6] Chapin J. R. (2000). Third-person perception and optimistic Bias among urban minority at-risk youth. Commun. Res. 27, 51–81. doi: 10.1177/009365000027001003

[ref7] Chen S. Tian X. (2024). What’s “positive” during Shanghai’s COVID-19 lockdown? Ideology, collectivism, and constructive journalism in China. Journal. Stud. 25, 703–722. doi: 10.1080/1461670x.2024.2331513

[ref8] Cheng Y. Luo Y. (2020). The presumed influence of digital misinformation: examining US public’s support for governmental restrictions versus corrective action in the COVID-19 pandemic. Online Inf. Rev. 45, 834–852. doi: 10.1108/oir-08-2020-0386

[ref9] Chua A. Y. K. Tee C.-Y. Pang A. Lim E.-P. (2017). The retransmission of rumor and rumor correction messages on twitter. Am. Behav. Sci. 61, 707–723. doi: 10.1177/0002764217717561

[ref10] Cinelli M. Quattrociocchi W. Galeazzi A. Valensise C. M. Brugnoli E. Schmidt A. L.. (2020). The COVID-19 social media infodemic. Sci. Rep. 10:16598. doi: 10.1038/s41598-020-73510-5, PMID: 33024152 PMC7538912

[ref11] Corbu N. Oprea D.-A. Negrea-Busuioc E. Radu L. (2020). ‘They can’t fool me, but they can fool the others!’ Third person effect and fake news detection. Eur. J. Commun. 35, 165–180. doi: 10.1177/0267323120903686

[ref12] Cotter K. DeCook J. R. Kanthawala S. (2022). Fact-checking the crisis: COVID-19, infodemics, and the platformization of truth. Soc. Media Soc. 8, 1–13. doi: 10.1177/20563051211069048

[ref13] Craig M. Vijaykumar S. (2023). One dose is not enough: the beneficial effect of corrective COVID-19 information is diminished if followed by misinformation. Soc. Media Soc. 9:20563051231161298. doi: 10.1177/20563051231161298, PMID: 37090481 PMC10111161

[ref14] Davison W. P. (1983). The third-person effect in communication. Public Opin. Q. 47, 1–15. doi: 10.1086/268763

[ref15] Duffy A. Tandoc E. Ling R. (2019). Too good to be true, too good not to share: the social utility of fake news. Inf. Commun. Soc. 23, 1965–1979. doi: 10.1080/1369118x.2019.1623904

[ref16] Ecker U. K. H. Lewandowsky S. Cook J. Schmid P. Fazio L. K. Brashier N.. (2022). The psychological drivers of misinformation belief and its resistance to correction. Nat. Rev. Psychol. 1, 13–29. doi: 10.1038/s44159-021-00006-y

[ref17] Eisend M. (2017). The third-person effect in advertising: a Meta-analysis. J. Advert. 46, 377–394. doi: 10.1080/00913367.2017.1292481

[ref18] Fleming N. (2020). Fighting coronavirus misinformation. Nature 583, 155–156. doi: 10.1038/d41586-020-01834-3, PMID: 32601491

[ref19] Fu J. Cook J. (2019). Browsing for Cunzaigan on WeChat: young People’s social media presence in accelerated urban China. Young 28, 404–421. doi: 10.1177/1103308819877787

[ref20] Garfin D. R. Silver R. C. Holman E. A. (2020). The novel coronavirus (COVID-2019) outbreak: amplification of public health consequences by media exposure. Health Psychol. 39, 355–357. doi: 10.1037/hea0000875, PMID: 32202824 PMC7735659

[ref21] Gerber J. P. Wheeler L. Suls J. (2018). A social comparison theory meta-analysis 60+ years on. Psychol. Bull. 144, 177–197. doi: 10.1037/bul0000127, PMID: 29144145

[ref22] Gollust S. E. Nagler R. H. Fowler E. F. (2020). The emergence of COVID-19 in the US: a public health and political communication crisis. J. Health Polit. Policy Law 45, 967–981. doi: 10.1215/03616878-8641506, PMID: 32464658

[ref23] Greifeneder R. Jaffe M. Newman E. Schwarz N. (2020). The psychology of fake news: Accepting, sharing, and correcting misinformation. London: Routledge.

[ref24] Gunther A. (1991). What we think others think: cause and consequence in the third-person effect. Commun. Res. 18, 355–372. doi: 10.1177/009365091018003004

[ref25] Hayes A. F. (2021). Introduction to mediation, moderation, and conditional process analysis: a regression-based approach. New York, NY: The Guilford Press.

[ref26] Johnson J. A. (2005). Ascertaining the validity of individual protocols from web-based personality inventories. J. Res. Pers. 39, 103–129. doi: 10.1016/j.jrp.2004.09.009

[ref27] Koo A. Z.-X. Su M.-H. Lee S. Ahn S.-Y. Rojas H. (2021). What motivates people to correct misinformation? Examining the effects of third-person perceptions and perceived norms. J. Broadcast. Electron. Media 65, 111–134. doi: 10.1080/08838151.2021.1903896

[ref28] Li X. (2008). Third-person effect, optimistic bias, and sufficiency resource in internet use. J. Commun. 58, 568–587. doi: 10.1111/j.1460-2466.2008.00400.x

[ref29] Li Y. Chandra Y. Kapucu N. (2020). Crisis coordination and the role of social Media in Response to COVID-19 in Wuhan, China. Am. Rev. Public Adm. 50, 698–705. doi: 10.1177/0275074020942105

[ref30] Meade A. W. Craig S. B. (2012). Identifying careless responses in survey data. Psychol. Methods 17, 437–455. doi: 10.1037/a0028085 22506584

[ref31] Mehrabian A. Russell J. A. (1974). An approach to environmental psychology. Cambridge, MA: MIT Press.

[ref32] Meirick P. C. (2002). Self-enhancement, self-affirmation and threats to self -worth: three tests of a motivational explanation for first- and third -person effects. Doctoral Dissertation. Minnesota. BTW: University of Minnesota.

[ref33] Nam B. H. Weber H. L. Liu Y. English A. S. (2022). The 'Myth of zero-COVID' nation: a digital ethnography of expats’ survival amid Shanghai lockdown during the omicron variant outbreak. Int. J. Environ. Res. Public Health 19:9047. doi: 10.3390/ijerph19159047, PMID: 35897419 PMC9332489

[ref34] Nathanson A. I. Eveland W. P. Park H. S. Paul B. (2002). Perceived media influence and efficacy as predictors of caregivers’ protective behaviors. J. Broadcast. Electron. Media 46, 385–410. doi: 10.1207/s15506878jobem4603_5

[ref35] Park H. S. Salmon C. T. (2005). A test of the third-person effect in public relations: application of social comparison theory. J. Mass Commun. Q. 82, 25–43. doi: 10.1177/107769900508200103

[ref36] Reyes L. M. Ortiz L. Abedi M. Luciano Y. Ramos W. Reyes P. J. J. (2021). Misinformation on COVID-19 origin and its relationship with perception and knowledge about social distancing: a cross-sectional study. PLoS One 16:e0248160. doi: 10.1371/journal.pone.0248160, PMID: 33690685 PMC7942988

[ref37] Rojas H. (2010). "Corrective" actions in the public sphere: how perceptions of media and media effects shape political behaviors. Int. J. Public Opinion Res. 22, 343–363. doi: 10.1093/ijpor/edq018

[ref38] Rojas H. Barnidge M. Abril E. P. (2016). Egocentric publics and corrective action. Commun. Public 1, 27–38. doi: 10.1177/2057047315619421

[ref39] Rosseel Y. (2012). Lavaan: AnRPackage for structural equation modeling. J. Stat. Softw. 48, 1–36. doi: 10.18637/jss.v048.i02

[ref40] Schmierbach M. Boyle M. P. McLeod D. M. (2008). Understanding person perceptions: comparing four common statistical approaches to third-person research. Mass Commun. Soc. 11, 492–513. doi: 10.1080/15205430802375311

[ref41] Shen C. Gong H. (2019). Personal ties, group ties and latent ties: connecting network size to diversity and trust in the mobile social network WeChat. Asian J. Commun. 29, 18–34. doi: 10.1080/01292986.2018.1504976

[ref42] Shi W.-z. Zeng F. Zhang A. Tong C. Shen X. Liu Z.. (2022). Online public opinion during the first epidemic wave of COVID-19 in China based on Weibo data. Hum. Soc. Sci. Commun. 9, 1–10. doi: 10.1057/s41599-022-01181-w

[ref43] Song H. So J. Shim M. Kim J. Kim E. Lee K. (2023). What message features influence the intention to share misinformation about COVID-19 on social media? The role of efficacy and novelty. Comput. Hum. Behav. 138:107439. doi: 10.1016/j.chb.2022.107439, PMID: 35974879 PMC9371473

[ref44] Stockmann D. Luo T. (2017). Which social media facilitate online public opinion in China? Problems Post-Communism 64, 189–202. doi: 10.1080/10758216.2017.1289818

[ref45] Su Y. Venkat A. Yadav Y. Puglisi L. B. Fodeh S. J. (2021). Twitter-based analysis reveals differential COVID-19 concerns across areas with socioeconomic disparities. Comput. Biol. Med. 132:104336. doi: 10.1016/j.compbiomed.2021.104336, PMID: 33761419 PMC9159205

[ref46] Suls J. Martin R. Wheeler L. (2002). Social comparison: why, with whom, and with what effect? Curr. Dir. Psychol. Sci. 11, 159–163. doi: 10.1111/1467-8721.00191

[ref47] Sun Y. Pan Z. Shen L. (2008a). Understanding the third-person perception: evidence from a Meta-analysis. J. Commun. 58, 280–300. doi: 10.1111/j.1460-2466.2008.00385.x

[ref48] Sun Y. Shen L. Pan Z. (2008b). On the behavioral component of the third-person effect. Commun. Res. 35, 257–278. doi: 10.1177/0093650207313167

[ref49] Talwar S. Dhir A. Singh D. Virk G. S. Salo J. (2020). Sharing of fake news on social media: application of the honeycomb framework and the third-person effect hypothesis. J. Retail. Consum. Serv. 57:102197. doi: 10.1016/j.jretconser.2020.102197

[ref50] Taylor L. (2022). Covid-19: China installs fences and alarms in Shanghai in effort to curb cases. BMJ 377:o1076. doi: 10.1136/bmj.o1076, PMID: 35477680

[ref51] Tsay-Vogel M. (2016). Me versus them: third-person effects among Facebook users. New Media Soc. 18, 1956–1972. doi: 10.1177/1461444815573476

[ref52] van der Meer T. G. Brosius A. Hameleers M. (2022). The role of media use and misinformation perceptions in optimistic Bias and third-person perceptions in times of high media dependency: evidence from four countries in the first stage of the COVID-19 pandemic. Mass Commun. Soc. 26, 438–462. doi: 10.1080/15205436.2022.2039202

[ref53] Verma P. (2022). Locked down, Shanghai residents skirt censorship to vent online. The Washington Post April 22th. https://www.washingtonpost.com/world/2022/04/22/shanghai-lockdown-social-media-posts/

[ref54] Vosoughi S. Roy D. Aral S. (2018). The spread of true and false news online. Science 359, 1146–1151. doi: 10.1126/science.aap9559 29590045

[ref55] Wang Q. Dai R. Zhang T. Li J. Sheng T. Wu B. (2022). Supply of basic necessities to vulnerable populations during the COVID-19 pandemic: empirical evidence from Shanghai, China. Front. Public Health 10:1008180. doi: 10.3389/fpubh.2022.1008180, PMID: 36388370 PMC9645813

[ref56] Wang R. Zhang H. (2023). Who spread COVID-19 (mis)information online? Differential informedness, psychological mechanisms, and intervention strategies. Comput. Hum. Behav. 138:107486. doi: 10.1016/j.chb.2022.107486, PMID: 36120514 PMC9467818

[ref57] Yang W. (2022). Shanghai residents expose life during lockdown. Deutsche Welle May 4th. https://www.dw.com/en/coronavirus-shanghai-residents-evade-censors-to-expose-life-during-lockdown/a-61683680

[ref58] Yang F. Horning M. (2020). Reluctant to share: how third person perceptions of fake news discourage news readers from sharing “real news” on social media. Soc. Media Soc. 6, 1–11. doi: 10.1177/2056305120955173

[ref59] Yang J. Tian Y. (2021). "Others are more vulnerable to fake news than I am": third-person effect of COVID-19 fake news on social media users. Comput. Hum. Behav. 125:106950. doi: 10.1016/j.chb.2021.106950, PMID: 35228774 PMC8867061

[ref60] Zhang B. Lei S. M. Le S. Gong Q. Cheng S. Wang X. (2022). Changes in health behaviors and conditions during COVID-19 pandemic strict campus lockdown among Chinese university students. Front. Psychol. 13:1022966. doi: 10.3389/fpsyg.2022.1022966, PMID: 36324783 PMC9621116

[ref61] Zhang X. Zhang W. Chen S. (2022). Shanghai’s life-saving efforts against the current omicron wave of the COVID-19 pandemic. Lancet 399, 2011–2012. doi: 10.1016/s0140-6736(22)00838-8, PMID: 35533708 PMC9075855

[ref62] Zhu Y. Wei R. Lo V.-H. Zhang M. Li Z. (2021). Collectivism and altruistic behavior: a third-person effect study of COVID-19 news among Wuhan residents. Global Media China 6, 476–491. doi: 10.1177/20594364211045568

